# Comparison of Proximal and Remote Sensing for the Diagnosis of Crop Status in Site-Specific Crop Management

**DOI:** 10.3390/s22010019

**Published:** 2021-12-22

**Authors:** Jiří Mezera, Vojtěch Lukas, Igor Horniaček, Vladimír Smutný, Jakub Elbl

**Affiliations:** Department of Agrosystems and Bioclimatology, Faculty of AgriSciences, Mendel University in Brno, Zemedelska 1, 61300 Brno, Czech Republic; jiri.mezera@mendelu.cz (J.M.); igor.horniacek@mendelu.cz (I.H.); vladimir.smutny@mendelu.cz (V.S.)

**Keywords:** remote sensing, N crop sensor, ISARIA, Sentinel, nitrogen, variable rate application

## Abstract

The presented paper deals with the issue of selecting a suitable system for monitoring the winter wheat crop in order to determine its condition as a basis for variable applications of nitrogen fertilizers. In a four-year (2017–2020) field experiment, 1400 ha of winter wheat crop were monitored using the ISARIA on-the-go system and remote sensing using Sentinel-2 multispectral satellite images. The results of spectral measurements of ISARIA vegetation indices (IRMI, IBI) were statistically compared with the values of selected vegetation indices obtained from Sentinel-2 (EVI, GNDVI, NDMI, NDRE, NDVI and NRERI) in order to determine potential hips. Positive correlations were found between the vegetation indices determined by the ISARIA system and indices obtained by multispectral images from Sentinel-2 satellites. The correlations were medium to strong (r = 0.51–0.89). Therefore, it can be stated that both technologies were able to capture a similar trend in the development of vegetation. Furthermore, the influence of climatic conditions on the vegetation indices was analyzed in individual years of the experiment. The values of vegetation indices show significant differences between the individual years. The results of vegetation indices obtained by the analysis of spectral images from Sentinel-2 satellites varied the most. The values of winter wheat yield varied between the individual years. Yield was the highest in 2017 (7.83 t/ha), while the lowest was recorded in 2020 (6.96 t/ha). There was no statistically significant difference between 2018 (7.27 t/ha) and 2019 (7.44 t/ha).

## 1. Introduction

Precision agriculture is a modern way of farming that adapts crop management practices to the heterogeneity of the soil condition. The main goal is to address the field-specific spatial variability of soil properties, microclimate conditions, crop vigor and crop yields. The development of this crop management system is strongly connected to the progression in agricultural technology, such as Global Positioning System (GPS), Geographic Information Systems (GIS), Remote Sensing (RS), soil and crop sensors and more [1,2]. The main advantage of precision agriculture is the efficient use of material inputs such as pesticides, mineral fertilizers, seeds and fuels according to the requirements of plants at the particular place and at the right time [3,4].

One of the integral parts of precision agriculture is variable or tagged nitrogen fertilization. The nutrition with nitrogen (N) (amide, ammonium and nitrate N) is the most important factor that affects the formation of yield and grain quality in cereals [5,6]. The general aim is to provide the plants sufficient N nutrition at the time of its need and to prevent its leaching, which would lead to eutrophication of the environment. The yield of cereals consists of three basic components, namely: number of ears (or heads) per unit area, number of grains in the ear and the weight of 1000 seeds [7]. A higher N dose generally increases the crop yield and the number and size of grains [8] but also reduces Nitrogen Use Efficiency (NUE) and increases the amount of residual N in the soil, leading to the risk of nitrogen leaching into groundwater [9,10,11].

The solution is a variable (targeted) application of N fertilizers, which respects specific soil conditions, in the form of management zones, and plant nutritional status when distributing the fertilizer into the soil. Consequently, the higher NUE and the lower risk of nitrogen leakage into the environment can be expected [12]. It is based on spectral vegetation measurement (proximal and remote), soil sampling, soil condition mapping or yield mapping [13,14]. The spatial variability of crop yields can be influenced by many factors such as evapotranspiration [15], topographic attributes [16] or combined effects of soil fertility and weed control [17]. The study by Diacono et al. [13] provides an overview of tools and approaches of precision agriculture for nitrogen management according to environmental requirements. Spectral measurement to estimate the nutritional status of plants mostly uses vegetation indices derived from the remote sensing systems of the Earth [18] or from the ground-based on-the-go systems [19].

For remote sensing purposes, it most often uses data from Landsat 8, Sentinel 2 and other types of regularly provided satellite imagery of Earth’s surface recordings, making them an effective remote sensing technique for gathering information over a large area with a high frequency of repetition [20]. Sentinel-2 (2A and 2B) satellites are equipped with a multispectral sensor (MSI) containing 13 spectral bands, including a near-infrared band with a spatial resolution from 10 to 60 m, providing relevant information to support precision agriculture [21]. Images from Sentinel-2 satellites are publicly available for free through the Copernicus Open Access Hub with an average repetition rate of 5 days (2–3 days in mid-latitudes) at data processing levels L1C or L2A. This may be interesting for the processing of data time series and for the application in precision agriculture. Product Level-2A provides images of the Bottom of the Atmosphere (BOA) with surface reflectance [6,22].

Another applicable technology in agriculture is proximal sensing by the on-the-go crop sensors. These devices are installed directly on the machines and use the measurement of red and near-infrared (NIR) reflectance for real-time assessment of plant nitrogen status with the simultaneous application of nitrogen fertilizers by spreader or sprayer. The most well-known systems include: Yara N-sensor, Crop Circle and Trimble GreenSeeker [23]. The new generation of these devices, such as Fritzmeier ISARIA, combines on-the-go spectral measurement of the crop stand with soil productivity maps (map-overlay mode). As shown by Pedersen et al. [24], the combination of soil information with the diagnosis of plant nitrogen status by spectral measurement has brought the greatest economic benefits of variable rate application of nitrogen fertilizers.

The aim of the study is to compare the sensor measurement of vegetation status using the Fritzmeier ISARIA on-the-go sensor system with remote sensing using satellite images of Sentinel-2 in the perspective of sensitivity and usability for plant diagnosis in the site-specific crop management of winter wheat.

## 2. Materials and Methods

### 2.1. Study Area

**Figure 1:**
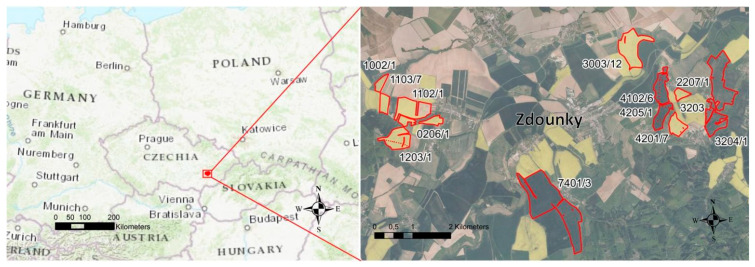
Localization of experimental fields. Individual experimental plots are marked with numerical codes.

**Figure 2:**
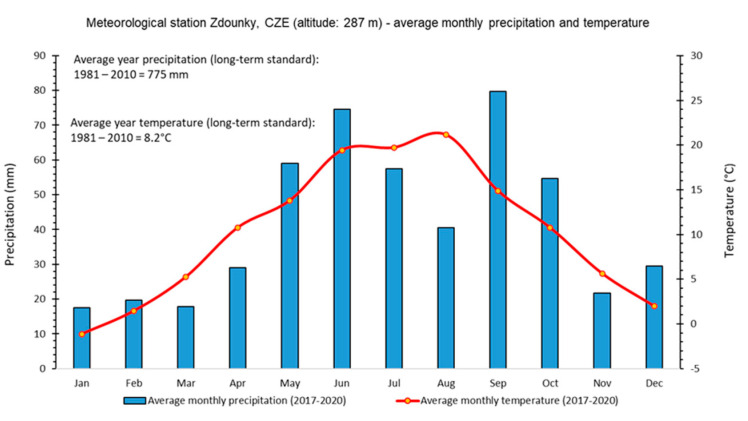
Climatic graph of the studied area (monthly precipitation and temperature): meteorological parameters for the period from 2017 to 2020 were measured by the DAVIS Vantage Pro2 meteorological station (Davis Instruments, Hayward, CA, USA). Long-term standards (1981–2010) for the Zdounky area were calculated on the basis of data available from the Czech Hydrometeorological Institute (http://portal.chmi.cz/historicka-data/, accessed 25 October 2021).

The selected localities mapped in the experiment are in the property of SALIX MORAVA Ltd. Company, Czech Republic. The agricultural company belongs to the Spearhead Czech Ltd. Business group (SIL CZ), which has been testing the technology of variable application of nitrogen fertilizers based on the use of sensor technology and remote sensing since 2012. This study contains data from four seasons (2017–2020) of growing winter wheat. The field experiment was established in the cadastral area of the municipality of Zdounky (Czech Republic, district Kromeriz, 49°13′ N, 17°18′ E; Figure 1). According to Quitt [25], climate in the studied region is slightly warm to warm and slightly damp (T3, MT2). The long-term average annual temperature was 8.2 °C in 1981–2010. The long-term average annual precipitation amount (1981–2010) was 775 mm (Figure 2). The fields are located at an altitude of 205–390 m a.s.l. Soil types are medium deep Chernozem, Haplic Luvisol, Cambisol and Fluvisol. The humus content is moderately high, equal to 2–3%. The soil pH ranges between 6.6 and 7.2. The fields are flat to moderately sloping.

The field experiment was performed from 2017 to 2020. Every year, min. 200 ha of arable land were selected (Table 1) to be sown with winter wheat (*Triticum aestivum* L.), which was a subject of the ISARIA system and satellite Sentinel-2. Winter wheat was cultivated using the conventional methods every year. Only the winter wheat intended for food purposes was monitored (2017: *Viriato*; 2018: *Dagmar*; 2019: *Viriato*; 2020: *Viriato*). The Viriato variety is legally protected and bred by Société RAGT 2n (FR) and the Dagmar variety by Limagrain Central Europe Cereals, s.r.o. (CZ, FR). These varieties can be defined as early bakery varieties suitable for milling purposes with similar growth and crop management properties. The term of sowing the winter wheat was from the last week of September to the first week of October depending on weather conditions. The average sowing rate of the model plant was 180 kg/ha, and the same system of N fertilization was adopted.

### 2.2. Crop Sensing

This study evaluates data from the ISARIA online sensor system and data from remote sensing using the Sentinel-2 satellite. The data were taken by the ISARIA sensor system (Table 1) in four vegetation seasons (2017–2020) in production fertilization (N2, BBCH 30–35). Satellite images were acquired during the entire vegetation period: For the assessment, one image was selected after the application of fertilizers in the given year. The image was selected to be cloudless in the studied locality and closest to the time of the ISARIA measurement. The statistical evaluation was focused on the identification of differences between the two sensing systems in terms of spectral sensitivity to vegetation and practical usage of the platforms. The study did not address the options of both sensing systems to set up nitrogen doses by the variable rate application, as it strongly depends on the algorithms delivered by the manufacturer (ISARIA) or service provider (remote sensing) and also specific user settings.

#### 2.2.1. Proximal Sensing by Fritzmeier ISARIA

**Figure 3:**
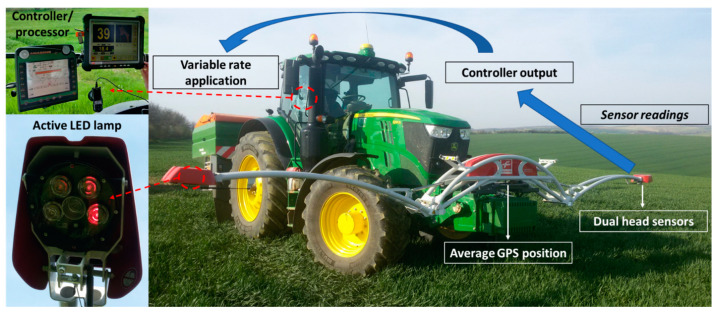
Nitrogen application by the Fritzmeier ISARIA crop sensing system in the field. Photo by J. Mezera (2019).

The recorded data were obtained from the Fritzmeier ISARIA proximal crop sensing system (Figure 3) during the second topdressing nitrogen application (N2). The device assesses the vegetation nutritional status based on optical measurement using two sensor heads sensing a space next to the trajectory of travel. The nutritional status is assessed by measuring the reflectivity of wheat, barley and rapeseed at a height between 40 and 100 cm from the crop by four active LEDs. The measured values are used for immediate recommendation of fertilizer dosing according to user settings and are recorded on the memory card.

The nutritional status of plants is evaluated by the spectral measurement of crops using active LED lighting at four spectral wavelengths (660–780 nm). Two vegetation indices are calculated—ISARIA Biomass Index (IBI), which is related to crop biomass and ISARIA Reflectance Measurement Index (IRMI) related to chlorophyll content. The IRMI index detects the nutritional status based on the reflectance of the red, NIR and red-edge spectral bands. The IBI index is based on the calculation of the reflectance in visible red and near-infrared radiation. The IBI index is used at earlier stages of development and, in addition to the amount of biomass, also evaluates the canopy of the stand [26].

The ISARIA system works in two calibration modes and an automatic mode with the simultaneous use of the background map of the yield potential to specify the production capacity of the site [27]. For the case of this study, the sensor worked in an absolute mode with the background map. ISARIA records were downloaded from the board computer as spatial point data in the shapefile format. Points at a distance of up to 20 m from the plot boundaries were filtered out of the dataset in order to avoid a possible impact on plot edges when bypassing obstacles, ramps and exits from the vegetation due to the elimination of boundary effects.

#### 2.2.2. Remote Sensing by Sentinel-2 Satellite Platform

Sentinel-2 images were selected to be cloudless and taken close to the date of proximal sensing by ISARIA. The time interval between ground and satellite sensing is presented in Table 1 by day in order to avoid the low-quality Sentinel-2 images. The longest time interval was 13 days in 2017, and the least difference was reached during N2 in 2018 (0–4 days).

The datasets were then downloaded from the ESA open hub database as a surface reflectance product L2A computed by sen2cor [28]. The low-resolution spectral bands of satellite imagery were resampled to 10 m per pixel by the Sentinel Application Platform (SNAP) provided by ESA [29], and the cloud mask was applied as a NoData pixel value based on the scene classification mask derived from the L2A dataset. A set of six vegetation indices was calculated from multispectral bands (see the list in Table 2) by the ArcPy (ESRI, Redlands, CA, USA) processing script to produce cloud-free 10 m resolution raster datasets [27].

### 2.3. Data Processing and Analysis

**Figure 4:**
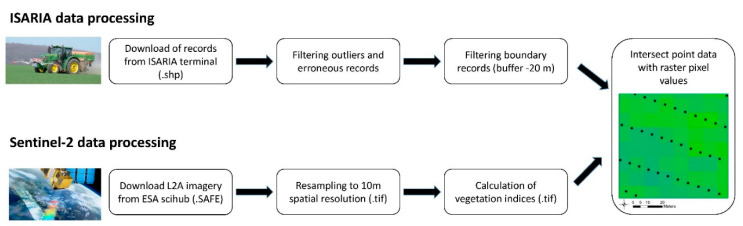
Flowchart of data processing and analysis in the study. Includes ISARIA point data and Sentinel-2 raster imagery pre-processing procedures in GIS and the overlay analysis of point and raster datasets.

For the given period of time, the value of the vegetation indices of the particular pixel of the Sentinel-2 satellite image was assigned to each point of the Frizmeier ISARIA online sensor after the final buffer by the overlay analysis. The flowchart of data processing of both sensing methods is shown in Figure 4. The spatial analysis of data, field identification, data merging and visualization of both datasets were realized in the Geographic Information System ArcMap 10.6.1 (ESRI, Redlands, CA, USA) in the coordinate system WGS 1984. While the spatial resolution of the Sentinel-2 imagery was 10 m per pixel after resampling of red-edge spectral bands, the spatial distribution of ISARIA records remained in the native resolution based on the 1 s acquisition and GPS position of tractor in the field.

Statistical evaluation (descriptive, regression and ANOVA) was carried out by Statistica 12 (Tibco, Palo Alto, CA, USA). All analyses were performed at a significance level of *p* < 0.05.

## 3. Results

The study aimed at a comparison of vegetation indices obtained from the ISARIA system (IBI, IRMI) with vegetation indices from the spectral analysis of satellite images taken by Sentinel-2 (EVI, GNDVI, NDMI, NDRE, NDVI, NRERI). Basic statistical data of both data sets for the period of 2017–2020 are presented in Table 3. A detailed overview of basic statistical data of vegetation indices of proximal and remote sensed data for individual years is shown in Annex 1. Each year, a minimum of 11,000 points were compared. In total, monitoring was carried out on more than 1400 ha of arable land with winter wheat (Table 1). The highest average values within the whole period of monitoring were reached by vegetation index IRMI and the second highest at IBI (Table 3). Both indices were counted using the ISARIA system within the application of production nitrogen dose to the winter wheat vegetation. Other indices (from satellite data from the same period) reached lower values. Maximum values reached 0.9 (NDVI, GNDVI), and the lowest values dropped below 0 (NRERI and NDMI). The order of the values was as follows: NDVI > GNDVI > EVI > NDRE > NDMI > NRERI.

**Figure 5:**
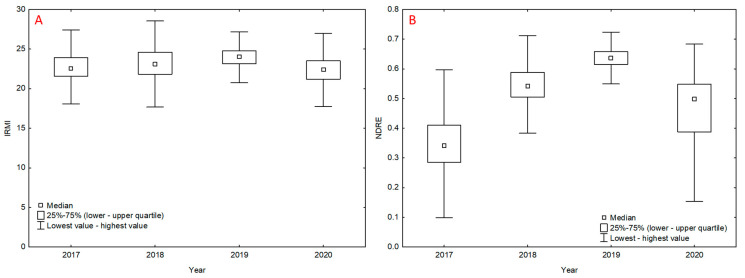
Average values of IRMI (part of graph (**A**)) and NDRE (part of graph (**B**)) vegetation indices in 2017–2020: Boxes show Quarter 1 (25%) and Quarter 3 (75%), red squares show the median and black brackets show minimal and maximal values.

**Figure A1:**
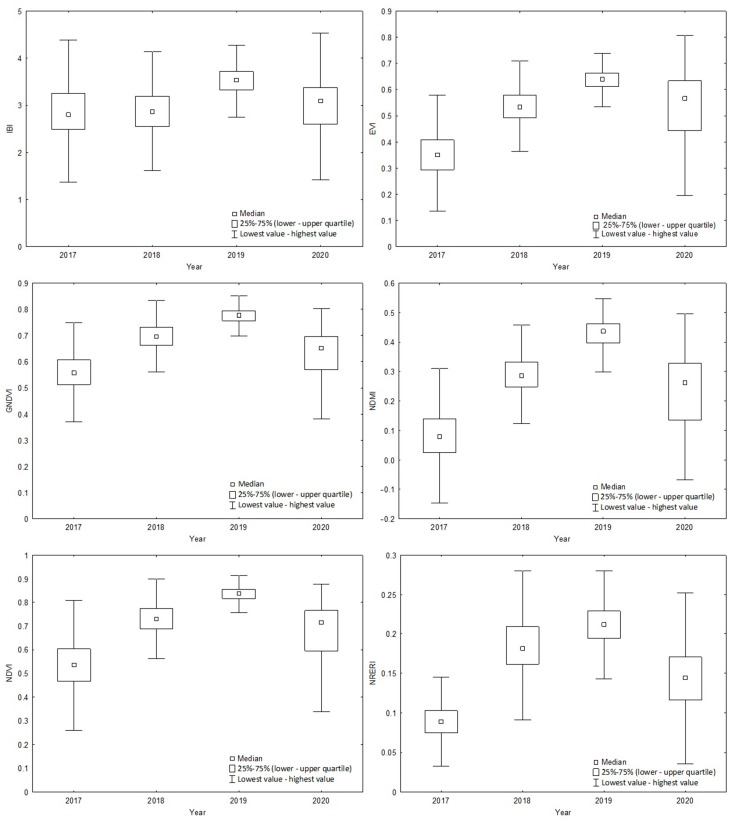
Vegetation indices for the individual years (2017, 2018, 2019, 2020): Boxes show Quarter 1 (25%) and Quarter 3 (75%), red squares show the median and black brackets show minimal and maximal values.

Selected vegetation indices were evaluated both for the whole vegetation period (2017–2020; Table 3) and for individual years (Appendix A; Table A1 and Figure A1). The measured effect shows the annual effect (Appendix A and Figure 5). The highest and lowest values for the vegetation indices were recorded in 2019 and 2017, respectively. The values of vegetation indices for individual years were subjected to post hoc analysis (Tukey’s HSD test), which revealed that all measured values showed significant differences between years (supplementary, Table S1). Therefore, the annual effect was significant for all variants. However, it was most pronounced in vegetation indices obtained by satellite image analysis. The measured data show (Figure 5) that the ISARIA vegetation indices showed lower relative differences between individual flights (e.g., IRMI, Figure 5A) compared to other vegetation indices (e.g., NDRE, Figure 5B).

The values of vegetation indices (Figure 5 and Figure A1) and values of winter wheat yield varied between individual years (Table 4). As to the development of the values of vegetation indices and the yield of the monitored crop, there was no conclusive relation. Yield was the highest in 2017, while the lowest yield was recorded in 2020. Between 2017 and 2018, the yield recorded a significant decrease as well as total precipitation totals (supplementary, Figure S1). In 2020, total precipitation amounts were at 82% of the long-term standard and yield was the lowest. The highest intensity of precipitation was recorded in June and July (supplementary, Figure S1), i.e., during the ripening and harvesting periods.

Furthermore, the relationship between the vegetation indices was analyzed using the correlation and regression analysis (Table 5, Table 6 and Table 7). The ISARIA vegetation indices were compared with the vegetation indices obtained by the spectral analysis of satellite images, both in terms of overall (total correlation) and individual images taken in particular years (Appendix B; Table A2 and Table A3). From the measured values (Table 5), it is evident that the IRMI vegetation index showed a positive correlation with all the other vegetation indices both in the individual years of the experiment and in general. It was the strongest against the IBI index, both in total correlation and when comparing data from the individual years of the experiment. Other vegetation indices showed a more variable dependence on IRMI, which differed both generally and in individual years. The strength of the relationship between IRMI and the other indices decreased as follows GNDVI > NDRE > NDVI > NDMI > NRERI in terms of overall correlation. Significant differences in the value of r were found within individual years. The highest one was recorded in 2020, when the vegetation indices always exceeded 0.77. The lowest values were recorded in 2017 (approximately 0.6).

The IBI vegetation index (Table 6) reached similar values of relation to the vegetation indices obtained by the satellite image analysis. The strongest correlation was again recorded in 2020, when the value of r exceeded the limit of 0.8 for all vegetation indices and, conversely, the lowest value of r was recorded in 2017.

**Figure 6:**
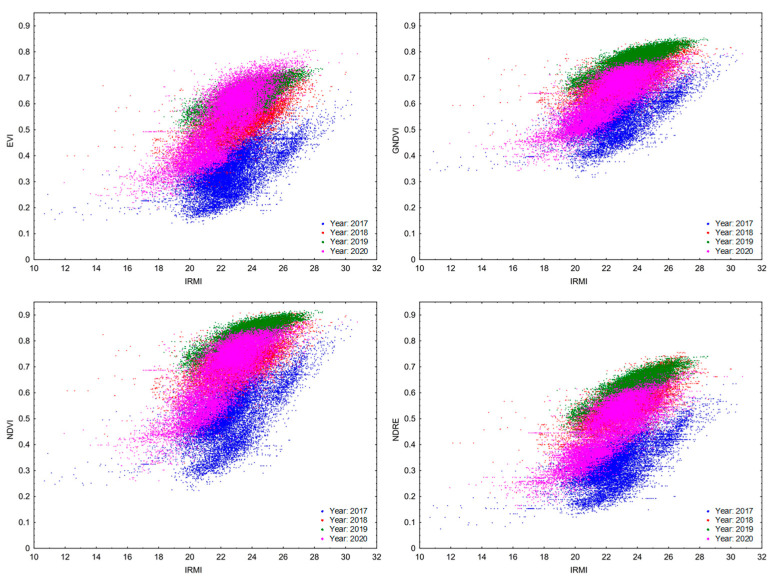
Spearman’s rank correlation coefficient and scatter graph showing the correlation between the IRMI vegetation index and the vegetation indices of EVI, GNDVI, NDRE and NDVI in the respective experimental years.

**Figure 7:**
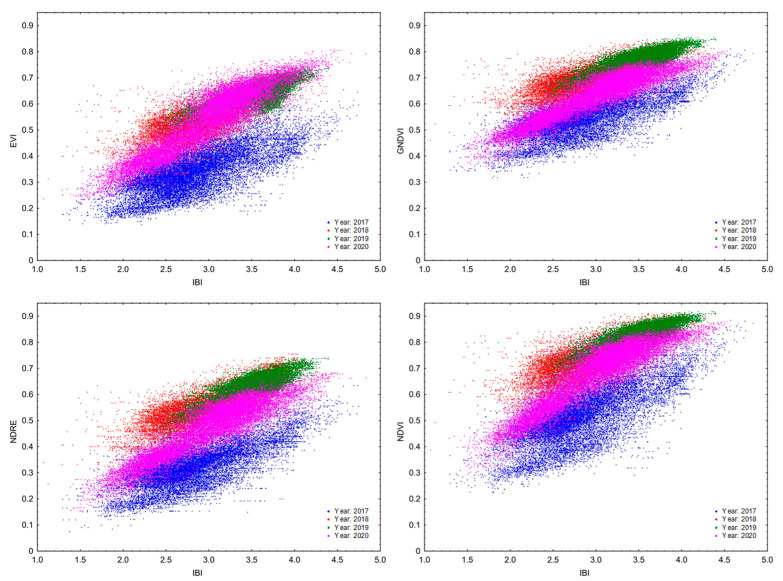
Spearman’s rank correlation coefficient and scatter graph showing the correlation between the IBI vegetation index and vegetation indexes EVI, GNDVI, NDRE and NDVI in the respective years of the experiment.

Selected correlations between the individual vegetation indices were analyzed. Table 7 shows a summary of regression equations, and Figure 5 and Figure 6 show the r values in the respective years. Correlations are displayed between the vegetation indices (vs. IRMI/IBI) whose r value was equal to or greater than 0.6 after the regression analysis. The graphs (Figure 6 and Figure 7) confirm the positive linear relationship between the vegetation indices of the ISARIA system and the indices (EVI, GNDVI, NDMI, NDRE, NDVI and NRERI) obtained by the spectral analysis of satellite images from Sentinel-2. From the overlap of r values in the individual years (2017–2020) and from the values of regression equations (Table 7), it is clear that there was a shift in the linear dependence, which indicates a potential annual effect (effect of total precipitation amounts and average temperatures in the respective years) on the monitored vegetation indices. The annual influence of meteorological conditions is also evident from the average grain yield from the individual plots (Table 4) and from the development of meteorological parameters in the individual years (supplementary, Figure S1).

**Figure 8:**
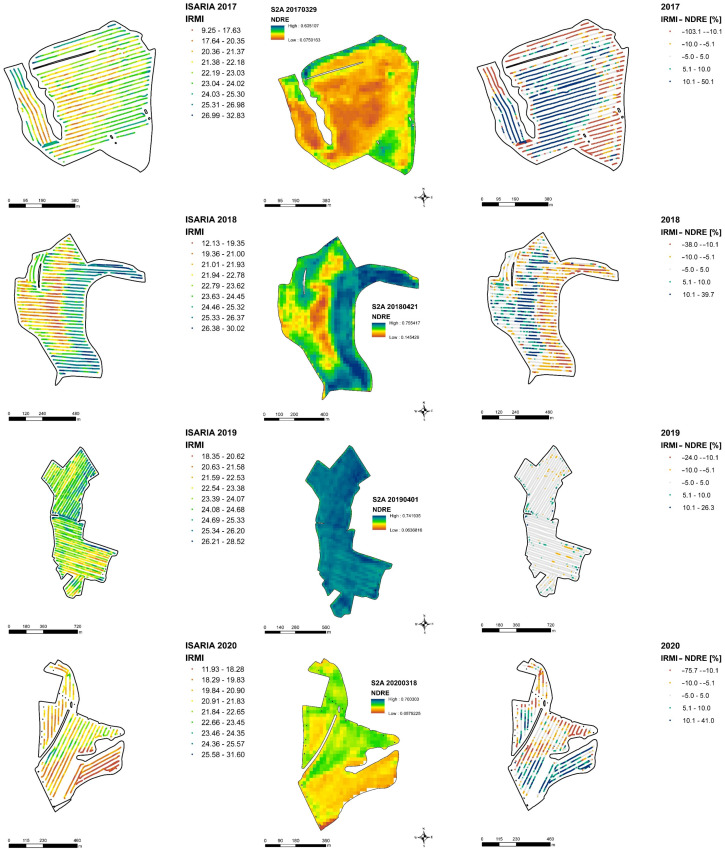
Example of the comparison of four experimental fields in the selected years: vegetation index ISARIA IRMI (**left**); vegetation index Sentinel-2 (**middle**); relative (%) comparison of differences between the two sensing systems (**right**).

Furthermore, examples of plots for each year are presented in Figure 8, the IRMI–ISARIA (left), and the NDRE–Sentinel-2 (middle) vegetation indices and a map of the relative comparison of both indices (right). The relative comparison maps show a comparison of two vegetation indices, and thus a comparison of mapping technologies. The maps were obtained by converting both indices to relative values. The calculation was carried out by using an average value of the particular index of the given plot and by subtracting the NDRE values from the IRMI index. The resulting maps are divided into five categories. The middle (gray) category shows the places on the map where both indices almost coincided within ± 5%. The yellow category shows the difference ranges from −10% to (−5)%, and the light green category is the difference range from 5% to 10%. Positive values (blue) indicate a category of difference higher than 10%, where a higher relative value of the IRMI index prevailed compared to the relative value of the NDRE index. Conversely, negative values (red) show a category of values lower than −10%, where higher relative values of the NDRE index prevailed as compared to relative values of the IRMI index.

The comparison of all plots (Table 8) between the individual years shows the highest agreement of technologies in 2019, when 75% of all values represented the category of ±5% and almost 97% of all values were indicated in the category of ±10%. The lowest values of conformity between the technologies were achieved in 2017, when 29% of values represented the ±5% category and 55% represented the ± 10% category.

**Figure A2:**
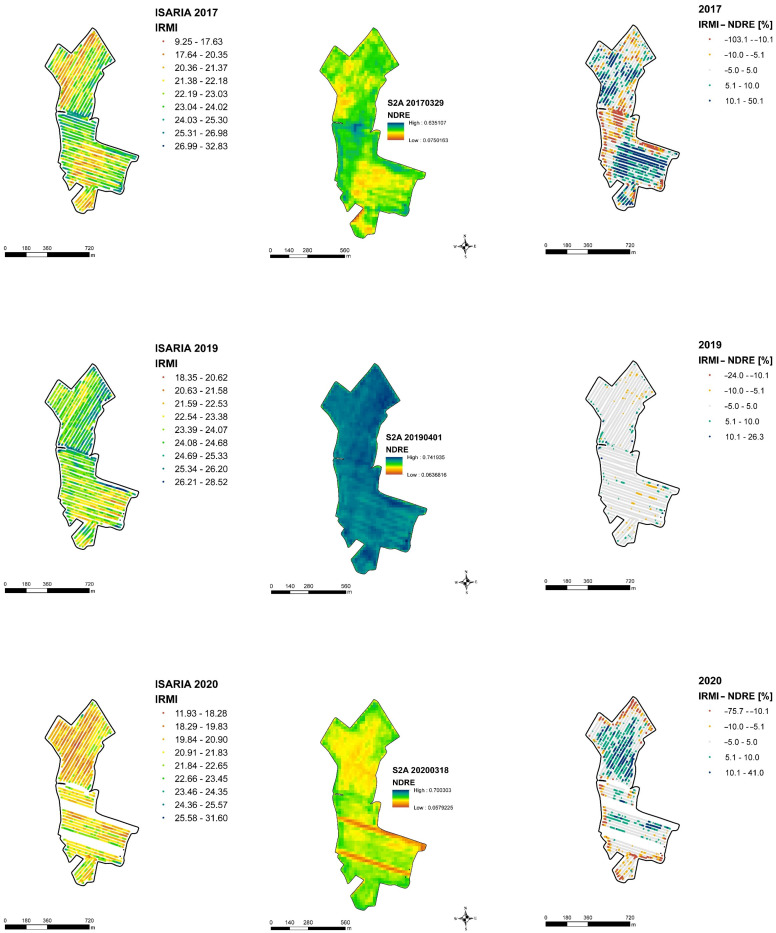
Example of the comparison of proximal and remote sensing on experimental field ID 2207_1 in the selected years: vegetation index ISARIA IRMI (**left**); vegetation index Sentinel-2 (**middle**); relative (%) comparison of differences between the two sensing systems (**right**).

**Figure A3:**
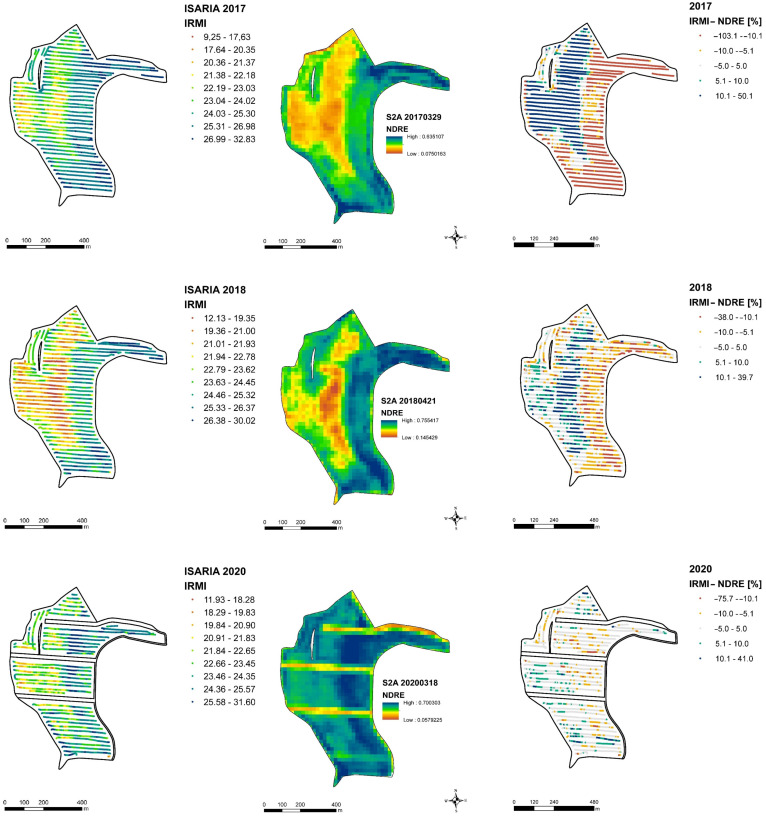
Example of the comparison of proximal and remote sensing on experimental field ID 3003_12 in the selected years: vegetation index ISARIA IRMI (**left**); vegetation index Sentinel-2 (**middle**); relative (%) comparison of differences between the two sensing systems (**right**).

**Figure A4:**
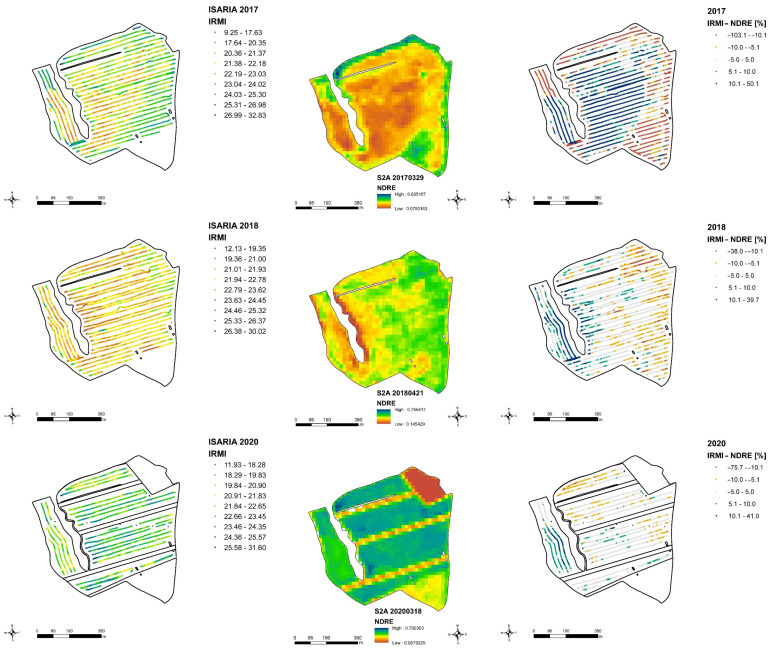
Example of the comparison of proximal and remote sensing on experimental field ID 3202_1 in the selected years: vegetation index ISARIA IRMI (**left**); vegetation index Sentinel-2 (**middle**); relative (%) comparison of differences between the two sensing systems (**right**).

On the maps of selected plots (Figure 8), we can see recurring trends over several years. In the places of higher absolute values of vegetation indices (IRMI, NDRE), negative relative values of the differences between the technologies are evident. It indicates that the Sentinel-2 satellite detected higher absolute values compared to the ISARIA system. In the places of lower absolute values of vegetation indices, the opposite effect is evident. On sites with visible areas of positive relative values of the difference, the ISARIA system detected higher absolute values than the Sentinel-2 satellite. In the case of Plot 1, these trends are seen in 2017, 2018 and 2020. In 2019, almost all values of the relative difference belong to the middle category ± 5%, which means that the results of both technologies almost coincided. An example of the relative comparison of selected plots in the respective years is included in the appendix (Appendix C, Figure A2, Figure A3 and Figure A4). In 2020, the bands in the plots are caused by the introduction of a new technology of erosion strips in the cultivated land of the company.

## 4. Discussion

The measured values confirm that the vegetation indices of the ISARIA system (IBI and IRMI) are positively correlated with the calculated vegetation indices EVI, GNDVI, NDRE, NDVI, NDMI and NRERI. Therefore, it can be stated that both vegetation indices IBI and IRMI and all the above-mentioned vegetation indices captured the same trends in the development of winter wheat vegetation. Similar results were obtained by Bausch and Khosla [36], who compared three vegetation indices from multispectral images of the commercial QuickBird satellite system to terrestrial optical measurements of the stand with the aim of determining the nutritional status of maize. Gozdowski et al. [37] describe the results of a similar study comparing a Landsat satellite survey with ground-based measurements using an AgLeader OptRx sensor. The dependence between the two monitoring systems was tighter on plots showing higher spatial variability, while the correlation was low on homogeneous plots.

From the measured values, it is evident that the positive correlation between the individual vegetation indices was influenced by a so-called annual influence, which can be characterized as seasonal changes in meteorological conditions. These changes are evident from the measured meteorological data (supplementary Materials—Figure S1), when, e.g., in 2018, a rapid decrease in total precipitation was recorded from a long-term (1981–2010) amount of 775 to 334 mm and to 477 mm in 2017. Development of plants was affected by water availability in the soil environment as it is known to be one of the factors influencing the development of the winter wheat plant [24,38]. Lack of water could have caused even changes in the chlorophyll content of the plants. According to Nikolaeva et al. [39], long-term drought stress reduces the water content in leaves. This results in changes in the chlorophyll content of wheat plants. At the beginning of the drought period, a slight increase in chlorophyll content was recorded, and then a decrease, but there were no changes in the ratio of chlorophyll a/b. If we take into account the time interval (8 days on average) between the imaging of the winter wheat stands by the ISARIA system and the Sentinel-2 satellites, results of changes in the chlorophyll content could have been affected by vegetation indices. In general, the chlorophyll content in the leaves (LCC) of winter wheat plants is used as an indicator of nutritional status and photosynthesis [40]. LCC in wheat leaves affects the spectral reflectance of the stand. A higher value of LCC content increases the reflectivity of NIR and decreases the reflectance of visible radiation. This is reflected in the resulting values of vegetation indices [41]. Therefore, changes in the LCC content due to drought could have affected the calculated vegetation indices. Another important factor is the period in which the spectral analysis of the stand was made (by using the ISARIA system or the Sentinel-2 satellites). In general, vegetation indices are the highest in the period of plant growth. Their values decrease after flowering and in the stage of ripening [42]. Fluctuations in total precipitation amounts in the experimental years could have had a considerable influence on the growth and ripening processes. Vegetation periods were shifted when the stands ripened earlier; this reduced the chlorophyll content in the plants and affected, as a final consequence, the calculated values of vegetation indices [43]. The spatial variability of plant status in the fields reflected differences in the soil properties, field topography and crop management. This also includes the variable application rates of P and K fertilizers applied on the studied fields (arable land) based on earlier observations and analyses (of soil, plants, etc.). Identification of the separate effects of these factors on the crop sensing records is very difficult; thus, only the spectral differences of both sensing techniques were evaluated.

A similar variability, as in the case of vegetation indices, was found in average yields, although the development of values (increase and decrease) did not reflect the values of vegetation indices. While in 2018, a significant decrease in the yield of winter wheat grain was detected, a significant increase in the vegetation indices was detected, as well as in the following years. These values can be explained mainly by the date of imaging/monitoring the stand, which was carried out in the period of production fertilization with N fertilizer. Thus, at least two months before the harvest, other abiotic and biotic factors could have had their impact on the yield. An example is the year 2020, which in terms of total rainfall does not indicate a problem of drought, compared to 2018. However, the problem was in the distribution of rainfall which was uneven, and precipitation was above average during the ripening and harvest of the monitored crops. This has led to the reduced bulk density of winter wheat and thus to the lower total yield [38,44].

The choice of vegetation index and sensor type is a very common factor in differences between remote and proximal sensing, as shown in a review study with the analysis of 66 scientific papers focused on monitoring maize [45]. After all, differences in the spectral configuration in the form of the number of spectral bands and their wavelengths are also manifested between the proximal sensors themselves [46]. In some cases, it is recommended to use a combination of both methods in the form of full-area mapping by remote sensing and ground measurement with a chlorophyll meter to detect N deficiency [47]. However, investment costs vary for the two technologies, as the main satellite imagery is available free of charge (or at a very low price) compared to the high purchasing price of crop sensors [48].

**Figure 9:**
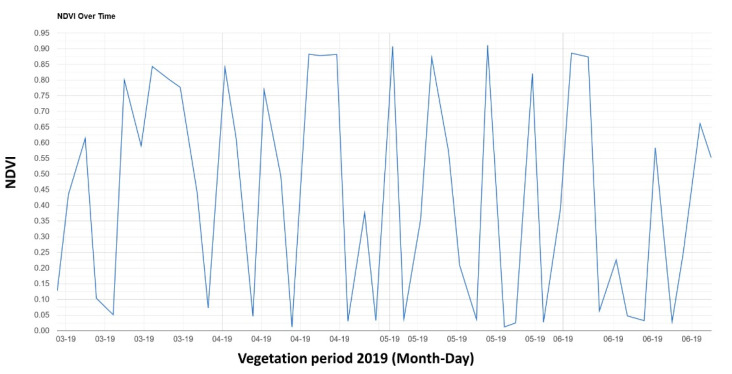
Overview of the cloud occurrence of Sentinel-2 images in the vegetation season 2019 (March–June) by indication of NDVI for locality Zdounky. Low NDVI values represent the occurrence of clouds in the observed scene (source: Google Earth Engine).

A demonstrable advantage of proximal sensing is operability/use in the case of increased cloudiness (Figure 9), which prevents a reliable use of remote sensing. Satellites cannot monitor the stand through cloudiness. The early and mid-growing season is typical for frequent cloudiness, which puts limits on the use of passive orbital sensing systems [49]. At the same time, proximal sensors can also be useful in case of the problematic evaluation of images of land areas near the treetops or objects that can distort the monitored stand by shading, e.g., trees. In areas with the frequent occurrence of clouds and in specific parts of the growing season, proximal sensors may represent a suitable alternative to remote sensing even beyond the monitoring of plant nutrition, e.g., even for the application of herbicides [50].

## 5. Conclusions

In this study, the optical measurements/assessments of vegetation by proximal and remote sensing methods were evaluated and compared for the on-farm diagnosis of plant nutritional status in site-specific crop management. The results of a four-year (2017–2020) field experiment showed a positive linear correlation between the vegetation indices obtained by the ISARIA proximal on-the-go sensing system (IRMI, IBI) and the indices determined by the spectral analysis of satellite images from the Sentinel-2 satellite (EVI, GNDVI, NDRE, NDVI, NDMI and NRERI). The dependence confirms that the compared vegetation in-dices are able to provide similar information on the condition of winter wheat during the growing season.

Positive correlations were found between vegetation indices determined by the ISARIA system and indices based on multispectral images from the Sentinel-2 satellites, which were moderately strong to strong (r = 0.54–0.81). Therefore, it can be stated that both technologies were able to capture a similar trend in the development of winter wheat vegetation. Furthermore, the influence of climatic conditions on the vegetation indices was analyzed between the individual years of the experiment. All vegetation indices demonstrated a significant effect of decreased total precipitation amounts and increased mean temperatures. The values of vegetation indices obtained by the analysis of spectral images from the Sentinel-2 satellites oscillated the most. This annual influence caused a change in the course of the linearization of the correlation. ISARIA vegetation indices showed lower differences among the individual years compared to the other vegetation indices (EVI, GNDVI, NDRE, NDVI, NDMI and NRERI). This effect was manifested by a shift in the linear dependence.

The results confirmed the similar sensitivity of proximal and remote crop sensing, their usability for the diagnosis of crop status, and their implementation for the variable application of nitrogen fertilizers during the vegetation period. The main difference between the two sensing methods, therefore, remains in their practical applicability. Sentinel-2 satellite data are available free of charge (or for a low operating fee) and represent a significant source of effective full-area vegetation mapping. However, a main disadvantage of satellite remote sensing is the risk of cloud and occurrence of other atmospheric phenomena in the scene, often with a higher frequency in the most important part of the growing season (April–May in the central European region). Just in these conditions, the proximal on-the-go sensors, such as ISARIA can be a suitable alternative for farm purposes despite their higher purchasing price.

## Figures and Tables

**Table 1:** Field experiment overview.

Year	ISARIA Sensing Date	Sentinel-2 Acquisit.	Days Difference	Area (ha)	Fields	Number of Records (*n*) Raw/Filtered	
2017	3–11 April	29 March	5–13	477	14	39,819	20,358
2018	17–25 April	21 April	0–4	211	9	24,951	11,597
2019	21 March–5 April	1 April	0–11	259	5	25,880	16,594
2020	17–26 March	18 March	0–8	439	14	40,011	19,655

**Table 2:** Sentinel-2 vegetation indices evaluated in the study.

Vegetation Index		Equation	Reference
EVI	Enhanced Vegetation Index	2.5 × (B08 − B04)/((B08 + 6.0 × B04 − 7.5 × B02) + 1.0)	Huete et al., (2002) [30]
GNDVI	Green Normalized Difference Vegetation Index	(B08 − B03)/(B08 + B03)	Gitelson et al., (1996) [31]
NDMI	Normalized Difference Moisture Index	(B08 − B11)/(B08 + B11)	Gao (1996) [32]
NDRE	Normalized Difference Red Edge Index	(B07 − B05)/(B07 + B05)	Barnes et al., (2000) [33]
NDVI	Normalized Difference Vegetation Index	(B08 − B04)/(B08 + B04)	Rouse et al., (1974) [34]
NRERI	Normalized Red Edge Index	(B08 − B06)/(B08 + B05)	Klem et al., (2014) [35]

**Table 3:** Results of descriptive statistics—vegetation indices for the period 2017–2020.

Variable	N	Average	MED	Min	Max	VAR	SD	VC
IRMI	68,204	23.001	23.017	10.667	30.750	3.442	1.855	8.065
IBI	68,204	3.079	3.105	1.070	4.830	0.300	0.548	17.788
EVI	68,204	0.507	0.531	0.137	0.807	0.019	0.137	27.107
GNDVI	68,204	0.656	0.669	0.317	0.865	0.011	0.104	15.859
NDMI	68,204	0.246	0.263	−0.165	0.547	0.024	0.155	62.902
NDRE	68,204	0.486	0.510	0.075	0.755	0.018	0.135	27.691
NDVI	68,204	0.683	0.716	0.223	0.918	0.021	0.143	20.994
NRERI	68,204	0.152	0.153	−0.011	0.347	0.003	0.057	37.380

Legend: N = number of measurements; MED = median; VAR = variance; SD = standard deviation; VC = variance coefficient; IRMI, IBI = ISARIA vegetation indices; EVI, GNDVI, NDMI, NDRE, NDVI, NRERI = Sentinel-2 vegetation indices.

**Table 4:** Results of descriptive statistics—vegetation indices for the period 2017–2020.

Year	Yield (t/ha)	±SD	HSD
2017	7.83	0.35	a
2018	7.27	0.19	b
2019	7.44	0.22	b
2020	6.96	0.27	c

Legend: average yield of winter wheat is displayed for the period 2017–2020 (*n* = 42) ± SD (Standard Deviation). Different small letters indicate Highly Significant Differences (HSD) between the individual years at a level of significance *p* < 0.05 (post hoc Tukey’s HSD test).

**Table 5:** Spearman’s rank correlation coefficient for the IRMI vegetation index in the individual years and for the whole period (2017–2020).

IRMI	IBI	EVI	GNDVI	NDMI	NDRE	NDVI	NRERI
Overall correlation	0.889	0.544	0.608	0.549	0.592	0.585	0.537
2017	0.919	0.608	0.602	0.581	0.624	0.599	0.511
2018	0.954	0.711	0.709	0.726	0.728	0.630	0.747
2019	0.913	0.614	0.676	0.540	0.707	0.671	0.652
2020	0.908	0.775	0.771	0.775	0.761	0.766	0.695

Legend: Red-marked Spearman’s correlation coefficients indicate a significant relationship between IRMI and other vegetation indices. The strength of this relationship is indicated using colors, red color of the cell indicates a weak relationship, orange and yellow indicate a moderate relationship and green indicates a strong relationship.

**Table 6:** Spearman’s rank correlation coefficient for the vegetation index IBI in the individual years and for the whole period (2017–2020).

IBI	IRMI	EVI	GNDVI	NDMI	NDRE	NDVI	NRERI
Overall correlation	0.889	0.708	0.719	0.689	0.703	0.729	0.622
2017	0.919	0.685	0.684	0.641	0.678	0.688	0.577
2018	0.954	0.733	0.745	0.759	0.765	0.692	0.752
2019	0.913	0.642	0.677	0.547	0.695	0.704	0.618
2020	0.908	0.889	0.890	0.886	0.875	0.892	0.819

Legend: Red-marked Spearman’s correlation coefficients indicate a significant relationship between IBI and other vegetation indices. The strength of this relationship is indicated using colors, the red color of the cells indicates a weak relationship, orange and yellow indicate a moderate relationship and green indicates a strong relationship.

**Table 7:** Regression equation of linear dependences.

Dependence	Equation
IRMI:EVI	y = 0.0379x − 0.3644
IRMI:GNDVI	y = 0.0334x − 0.1129
IRMI:NDMI	y = 0.0435x − 0.7543
IRMI:NDRE	y = 0.0414x − 0.466
IRMI:NDVI	y = 0.043x − 0.307
IRMI:NRERI	y = 0.0159x − 0.2128
IBI:EVI	y = 0.1716x − 0.0212
IBI:GNDVI	y = 0.1362x − 0.2365
IBI:NDMI	y = 0.1896x − 0.3376
IBI:NDRE	y = 0.1704x − 0.0388
IBI:NDVI	y = 0.1858x − 0.1109
IBI:NRERI	y = 0.0634x − 0.0435

**Table 8:** Percentage of relative differences between IRMI and NDRE in the individual years 2017–2020.

Year	Number of Records (*n*)	Category ± 5% (*n*)	Category ± 10% (*n*)	Category ± 5% (%)	Category ± 10% (%)
2017	20,358	5933	11,165	29.14	54.84
2018	11,597	6923	10,746	59.70	92.66
2019	16,594	12,496	16,032	75.30	96.61
2020	19,655	11,609	17,401	59.06	88.53

## Data Availability

Not applicable.

## Supplementary Materials

The following supplementary materials are available online at https://www.mdpi.com/article/10.3390/s22010019/s1, Table S1: Tukey HSD post hoc test for the main effect of years on the variables (vegetation indexes), Figure S1: Monthly precipitation and average temperature of our area of interest for individual years of field experiments.

## Appendix A. Results of Descriptive Statistics

**Table A1:** Results of descriptive statistics—vegetation indexes for individual years (2017, 2018, 2019, 2020).

Variable	Year	N	X	MED	MIN	MAX	VAR	SD	VC
IRMI	2017	20,358	22.851	22.550	10.667	30.383	3.670	1.916	8.384
IBI	2017	20,358	2.912	2.805	1.295	4.745	0.332	0.576	19.785
EVI	2017	20,358	0.351	0.351	0.137	0.655	0.006	0.080	22.835
GNDVI	2017	20,358	0.559	0.557	0.317	0.811	0.005	0.071	12.767
NDMI	2017	20,358	0.079	0.079	−0.165	0.378	0.006	0.080	102.016
NDRE	2017	20,358	0.345	0.341	0.075	0.635	0.007	0.085	24.709
NDVI	2017	20,358	0.537	0.536	0.223	0.884	0.011	0.107	19.907
NRERI	2017	20,358	0.091	0.089	−0.011	0.237	0.001	0.025	27.501
IRMI	2018	11,597	23.185	23.133	12.133	30.017	3.479	1.865	8.045
IBI	2018	11,597	2.897	2.870	1.070	4.375	0.201	0.448	15.481
EVI	2018	11,597	0.537	0.533	0.199	0.747	0.005	0.073	13.568
GNDVI	2018	11,597	0.699	0.696	0.408	0.851	0.003	0.057	8.088
NDMI	2018	11,597	0.295	0.286	0.004	0.534	0.005	0.074	25.071
NDRE	2018	11,597	0.548	0.542	0.214	0.755	0.005	0.071	12.860
NDVI	2018	11,597	0.732	0.729	0.329	0.911	0.005	0.073	9.988
NRERI	2018	11,597	0.187	0.182	0.060	0.315	0.001	0.038	20.237
IRMI	2019	16,594	23.864	24.017	18.350	28.517	1.982	1.408	5.899
IBI	2019	16,594	3.494	3.538	1.570	4.395	0.102	0.319	9.125
EVI	2019	16,594	0.635	0.640	0.432	0.745	0.002	0.043	6.702
GNDVI	2019	16,594	0.772	0.778	0.610	0.865	0.001	0.035	4.576
NDMI	2019	16,594	0.427	0.438	0.214	0.547	0.003	0.053	12.354
NDRE	2019	16,594	0.631	0.637	0.428	0.742	0.002	0.046	7.219
NDVI	2019	16,594	0.832	0.839	0.623	0.918	0.001	0.038	4.596
NRERI	2019	16,594	0.211	0.212	0.098	0.347	0.001	0.029	13.623
IRMI	2020	19,655	22.320	22.433	11.933	30.750	3.280	1.811	8.114
IBI	2020	19,655	3.007	3.095	1.385	4.830	0.293	0.542	18.008
EVI	2020	19,655	0.542	0.567	0.197	0.807	0.014	0.116	21.447
GNDVI	2020	19,655	0.633	0.653	0.343	0.802	0.007	0.081	12.858
NDMI	2020	19,655	0.238	0.263	−0.067	0.496	0.013	0.113	47.602
NDRE	2020	19,655	0.472	0.499	0.154	0.683	0.010	0.099	20.991
NDVI	2020	19,655	0.680	0.715	0.261	0.878	0.013	0.113	16.615
NRERI	2020	19,655	0.145	0.144	0.030	0.286	0.001	0.038	25.910

Legend: MED = median; X = average; VAR = variance; SD = standard deviation; VC = variance coefficient.

## Appendix B. Results of Regression Analysis

**Table A2:** Spearman’s rank correlation coefficient for the vegetation indexes IRMI within selected individual experimental fields and year.

Year	Exp. Field	Land Area (ha)	VC	IBI	EVI	GNDVI	NDMI	NDRE	NDVI	NRERI
2017	2207_1	58.21	8.44	0.95	0.66	0.69	0.73	0.84	0.61	0.40
2019	2207_1	58.21	3.85	0.91	0.70	0.71	0.65	0.67	0.69	0.73
2020	2207_1	54.62	4.99	0.78	0.50	0.59	0.55	0.56	0.55	0.44
2017	3204_1	29.56	10.61	0.82	0.67	0.70	0.66	0.71	0.68	0.53
2019	3204_1	29.56	5.28	0.95	0.80	0.85	0.81	0.80	0.83	0.78
2020	3204_1	29.28	9.22	0.92	0.79	0.85	0.83	0.86	0.83	0.56
2017	4102_6	24.75	4.78	0.89	0.64	0.63	0.64	0.61	0.63	0.45
2019	4102_6	24.75	5.15	0.78	0.61	0.68	0.70	0.68	0.67	0.63
2020	4102_6	22.57	5.15	0.91	0.71	0.70	0.68	0.72	0.69	0.58
2017	4205_1	18.16	5.52	0.96	0.75	0.73	0.69	0.71	0.74	0.55
2019	4205_1	17.94	3.31	0.78	0.67	0.67	0.65	0.66	0.68	0.60
2020	4205_1	15.7	5.43	0.91	0.81	0.79	0.76	0.77	0.79	0.67
2017	7401_3	128.32	8.00	0.97	0.69	0.71	0.61	0.72	0.75	0.62
2019	7401_3	128.32	4.59	0.87	0.38	0.47	0.30	0.54	0.43	0.52
2020	7401_3	114.86	5.07	0.91	0.73	0.73	0.73	0.74	0.69	0.72
2017	0206_1	8.97	5.59	0.84	0.64	0.68	0.70	0.73	0.68	0.64
2018	0206_1	8.97	4.20	0.96	0.49	0.55	0.62	0.64	0.50	0.63
2020	0206_1	9.22	4.66	0.92	0.33	0.40	0.39	0.34	0.43	0.48
2017	1002_1	7.46	3.39	0.94	0.63	0.56	0.62	0.66	0.58	0.46
2018	1002_1	7.96	4.17	0.97	0.56	0.44	0.50	0.50	0.47	0.47
2020	1002_1	7.97	4.36	0.91	0.60	0.57	0.57	0.55	0.59	0.48
2017	1102_1	44.7	4.68	0.93	0.73	0.71	0.71	0.73	0.72	0.65
2018	1102_1	37.3	5.03	0.96	0.65	0.65	0.70	0.72	0.64	0.65
2020	1102_1	42.63	4.57	0.84	0.52	0.48	0.50	0.48	0.45	0.51
2017	1103_7	11.13	3.92	0.85	0.62	0.60	0.52	0.54	0.59	0.52
2018	1103_7	11.13	3.95	0.97	0.63	0.73	0.79	0.79	0.72	0.80
2020	1103_7	11.13	9.31	0.95	0.72	0.77	0.78	0.79	0.77	0.69
2017	1203_1	29.81	5.83	0.95	0.74	0.74	0.69	0.75	0.76	0.63
2018	1203_1	29.81	4.54	0.93	0.65	0.62	0.67	0.65	0.61	0.60
2020	1203_1	27.88	3.96	0.92	0.61	0.65	0.60	0.62	0.65	0.55
2017	3003_12	45.12	8.01	0.99	0.91	0.91	0.92	0.93	0.93	0.83
2018	3003_12	45.12	9.46	0.98	0.88	0.86	0.88	0.88	0.86	0.87
2020	3003_12	40.16	6.62	0.95	0.71	0.73	0.75	0.76	0.72	0.75
2017	3202_1	46.11	6.60	0.98	0.79	0.76	0.79	0.77	0.77	0.59
2018	3202_1	46.11	4.90	0.91	0.42	0.47	0.50	0.52	0.43	0.36
2020	3202_1	38.11	3.83	0.81	0.44	0.48	0.44	0.53	0.48	0.48
2017	4201_7	16.89	4.75	0.98	0.84	0.80	0.82	0.80	0.81	0.58
2018	4201_7	16.89	4.16	0.84	0.40	0.37	0.45	0.43	0.31	0.38
2020	4201_7	16.89	3.44	0.82	0.51	0.49	0.58	0.63	0.42	0.57
2017	3203	7.71	5.58	0.98	0.70	0.63	0.68	0.61	0.63	0.52
2018	3203	7.71	4.57	0.91	0.64	0.69	0.69	0.72	0.70	0.60
2020	3203	7.71	4.10	0.89	0.50	0.50	0.50	0.51	0.48	0.48

Legend: VC = variance coefficient. Significant values are indicated by red marks.

**Table A3:** Spearman’s rank correlation coefficient for the IBI vegetation indices in the selected individual experimental fields and year.

Year	Exp. Field	Land Area (ha)	VC	IRMI	EVI	GNDVI	NDMI	NDRE	NDVI	NRERI
2017	2207_1	58.21	18.14	0.95	0.72	0.75	0.78	0.88	0.68	0.45
2019	2207_1	58.21	5.79	0.91	0.65	0.73	0.66	0.67	0.74	0.70
2020	2207_1	54.62	11.06	0.78	0.70	0.82	0.79	0.79	0.82	0.59
2017	3204_1	29.56	16.76	0.82	0.73	0.72	0.72	0.70	0.74	0.55
2019	3204_1	29.56	8.47	0.95	0.84	0.87	0.83	0.82	0.86	0.80
2020	3204_1	29.28	15.04	0.92	0.84	0.86	0.84	0.84	0.87	0.58
2017	4102_6	24.75	12.21	0.89	0.78	0.77	0.77	0.71	0.78	0.59
2019	4102_6	24.75	10.25	0.78	0.74	0.76	0.76	0.71	0.77	0.71
2020	4102_6	22.57	9.65	0.91	0.72	0.75	0.73	0.76	0.77	0.56
2017	4205_1	18.16	14.60	0.96	0.80	0.77	0.76	0.76	0.79	0.53
2019	4205_1	17.94	8.20	0.78	0.61	0.80	0.80	0.77	0.79	0.71
2020	4205_1	15.7	12.99	0.91	0.87	0.87	0.87	0.81	0.88	0.68
2017	7401_3	128.32	18.38	0.97	0.75	0.77	0.67	0.77	0.81	0.65
2019	7401_3	128.32	6.30	0.87	0.46	0.53	0.39	0.59	0.51	0.56
2020	7401_3	114.86	8.71	0.91	0.73	0.74	0.73	0.75	0.72	0.72
2017	0206_1	8.97	11.50	0.84	0.64	0.75	0.70	0.73	0.76	0.67
2018	0206_1	8.97	7.09	0.96	0.43	0.48	0.54	0.56	0.46	0.57
2020	0206_1	9.22	7.20	0.92	0.33	0.45	0.41	0.36	0.50	0.46
2017	1002_1	7.46	8.52	0.94	0.68	0.64	0.69	0.72	0.67	0.50
2018	1002_1	7.96	8.48	0.97	0.58	0.45	0.50	0.51	0.48	0.48
2020	1002_1	7.97	11.88	0.91	0.73	0.73	0.75	0.74	0.75	0.59
2017	1102_1	44.7	11.09	0.93	0.81	0.80	0.80	0.80	0.81	0.70
2018	1102_1	37.3	9.28	0.96	0.64	0.64	0.69	0.71	0.65	0.62
2020	1102_1	42.63	10.31	0.84	0.73	0.72	0.73	0.71	0.70	0.67
2017	1103_7	11.13	8.21	0.85	0.66	0.60	0.57	0.52	0.66	0.42
2018	1103_7	11.13	6.63	0.97	0.65	0.69	0.75	0.74	0.68	0.77
2020	1103_7	11.13	18.49	0.95	0.79	0.79	0.82	0.81	0.81	0.72
2017	1203_1	29.81	15.21	0.95	0.79	0.76	0.72	0.76	0.80	0.64
2018	1203_1	29.81	9.19	0.93	0.62	0.61	0.65	0.66	0.62	0.56
2020	1203_1	27.88	9.30	0.92	0.67	0.74	0.65	0.67	0.74	0.60
2017	3003_12	45.12	16.95	0.99	0.89	0.90	0.90	0.91	0.91	0.81
2018	3003_12	45.12	18.79	0.98	0.88	0.87	0.89	0.89	0.88	0.86
2020	3003_12	40.16	9.86	0.95	0.75	0.74	0.77	0.75	0.74	0.76
2017	3202_1	46.11	18.52	0.98	0.78	0.77	0.79	0.77	0.78	0.59
2018	3202_1	46.11	9.94	0.91	0.34	0.41	0.43	0.45	0.40	0.28
2020	3202_1	38.11	6.77	0.81	0.57	0.63	0.60	0.62	0.65	0.52
2017	4201_7	16.89	12.12	0.98	0.85	0.81	0.82	0.80	0.83	0.57
2018	4201_7	16.89	8.60	0.84	0.21	0.23	0.28	0.29	0.23	0.23
2020	4201_7	16.89	5.39	0.82	0.51	0.61	0.54	0.58	0.60	0.56
2017	3203	7.71	13.30	0.98	0.74	0.66	0.71	0.63	0.67	0.55
2018	3203	7.71	10.39	0.91	0.56	0.61	0.62	0.65	0.63	0.52
2020	3203	7.71	6.67	0.89	0.62	0.64	0.64	0.57	0.63	0.55

Legend: VC = variance coefficient. Significant values are indicated by red marks.
