# Supplementary material for: Comparison of Proximal and Remote Sensing for the Diagnosis of Crop Status in Site-Specific Crop Management
Source: Sensors (Basel). 2021 Dec 22;22(1):19. doi: 10.3390/s22010019 (PMC8747194; doi:10.3390/s22010019)
Supplement: Supplementary file 1 [file sensors-22-00019-s001.zip › sensors-1452663-supplementary.pdf]

Supplement Materials

Table S1. Tukey HSD post-hoc test for the main effect of years on the variables (vegetation indexes).

IRMI					IBI				
Year	2017	2018	2019	2020	Year	2017	2018	2019	2020
2017		0.000008	0.000008	0.000008	2017		0.028327	0.000008	0.000008
2018	0.000008		0.000008	0.000008	2018	0.028327		0.000008	0.000008
2019	0.000008	0.000008		0.000008	2019	0.000008	0.000008		0.000008
2020	0.000008	0.000008	0.000008		2020	0.000008	0.000008	0.000008	
EVI					GNDVI				
2017		0.000008	0.000008	0.000008	2017		0.000008	0.000008	0.000008
2018	0.000008		0.000008	0.000015	2018	0.000008		0.000008	0.000008
2019	0.000008	0.000008		0.000008	2019	0.000008	0.000008		0.000008
2020	0.000008	0.000015	0.000008		2020	0.000008	0.000008	0.000008	
NDMI					NDRE				
2017		0.000008	0.000008	0.000008	2017		0.000008	0.000008	0.000008
2018	0.000008		0.000008	0.000008	2018	0.000008		0.000008	0.000008
2019	0.000008	0.000008		0.000008	2019	0.000008	0.000008		0.000008
2020	0.000008	0.000008	0.000008		2020	0.000008	0.000008	0.000008	
NDVI					NRERI				
2017		0.000008	0.000008	0.000008	2017		0.000008	0.000008	0.000008
2018	0.000008		0.000008	0.000008	2018	0.000008		0.000008	0.000008
2019	0.000008	0.000008		0.000008	2019	0.000008	0.000008		0.000008
2020	0.000008	0.000008	0.000008		2020	0.000008	0.000008	0.000008	

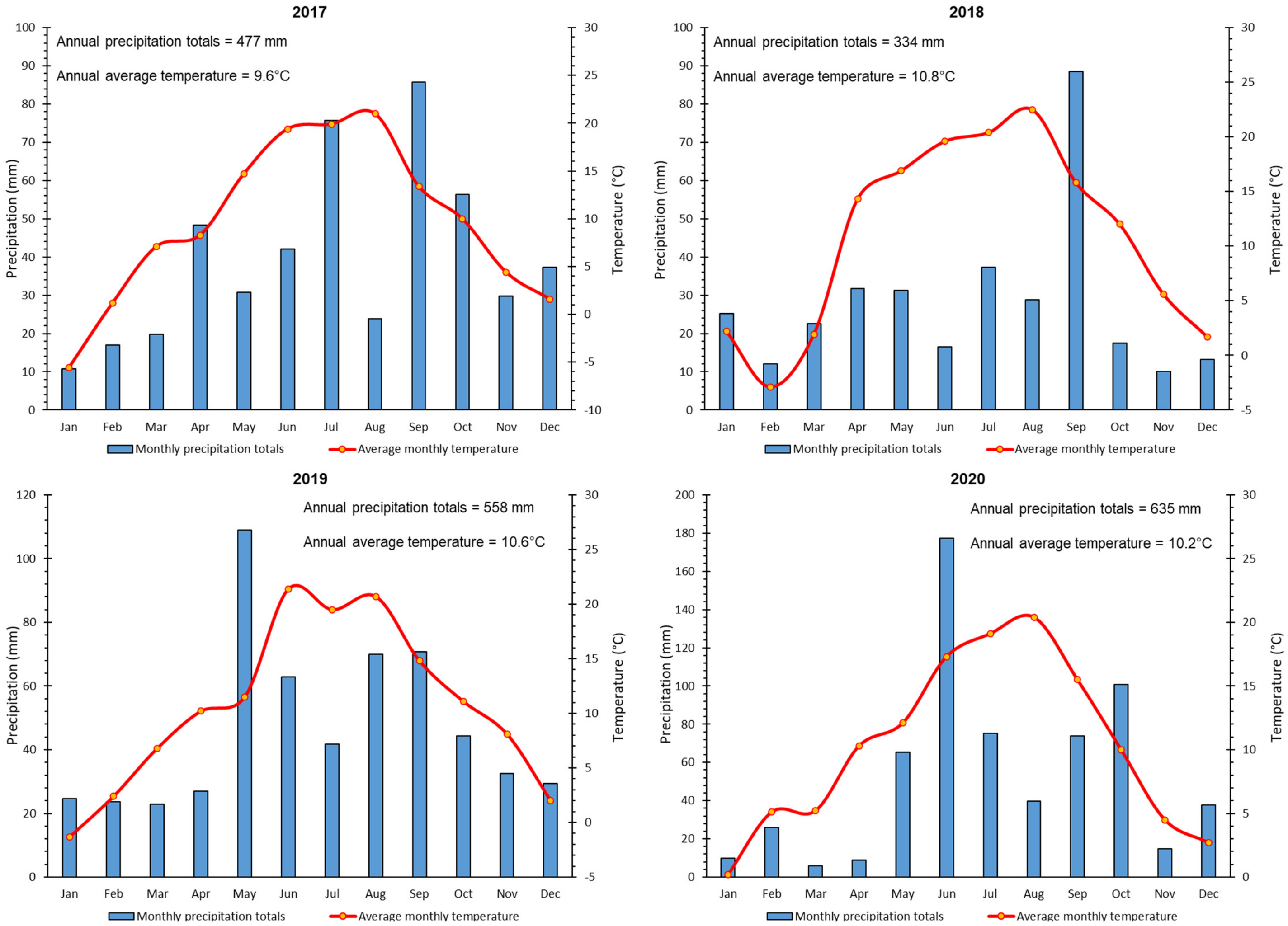

Figure S1. Monthly precipitation and average temperature of area of our interest for individual years of field experiment: Meteorological parameters for individual years (2017–2020) were measured by DAVIS Vantage Pro2 meteorological station (Davis Instruments, CA, USA).
